# Effects of Four Different Regulatory Mechanisms on the Dynamics of Gene Regulatory Cascades

**DOI:** 10.1038/srep12186

**Published:** 2015-07-17

**Authors:** Sabine Hansen, Sandeep Krishna, Szabolcs Semsey, Sine Lo Svenningsen

**Affiliations:** 1 Department of Biology, University of Copenhagen, Copenhagen, 2200, Denmark; 2 Simons Centre for the Study of Living Machines, National Centre for Biological Sciences, Bangalore, 560065, India; 3 Center for Models of Life, Niels Bohr Institute, University of Copenhagen, Copenhagen, 2100, Denmark

## Abstract

Gene regulatory cascades (GRCs) are common motifs in cellular molecular networks. A given logical function in these cascades, such as the repression of the activity of a transcription factor, can be implemented by a number of different regulatory mechanisms. The potential consequences for the dynamic performance of the GRC of choosing one mechanism over another have not been analysed systematically. Here, we report the construction of a synthetic GRC in *Escherichia coli*, which allows us for the first time to directly compare and contrast the dynamics of four different regulatory mechanisms, affecting the transcription, translation, stability, or activity of a transcriptional repressor. We developed a biologically motivated mathematical model which is sufficient to reproduce the response dynamics determined by experimental measurements. Using the model, we explored the potential response dynamics that the constructed GRC can perform. We conclude that dynamic differences between regulatory mechanisms at an individual step in a GRC are often concealed in the overall performance of the GRC, and suggest that the presence of a given regulatory mechanism in a certain network environment does not necessarily mean that it represents a single optimal evolutionary solution.

The function of living cells is controlled by complex regulatory networks that are built of molecular components of diverse chemical nature. Such networks ensure that gene products, such as enzymes, structural proteins, and RNA molecules, are synthesized when they are needed and in proper amounts. Gene regulatory cascades (in which one regulator controls the amount or activity of a second regulator, which controls a third regulator, and so on) are common motifs in these networks. Diverse mechanisms are available for controlling the different steps of transcription and translation, and the activity and stability of the produced protein in these cascades^1^,^2^,^3^,^4^,^5^,^6^. Several regulatory mechanisms have been studied in detail, i.e. the structure of the molecules involved and their specific interactions are characterized. Recently, some regulatory systems have also been studied as parts of small synthetic networks or embedded into the intracellular regulatory network, both experimentally and theoretically^7^,^8^,^9^,^10^,^11^. These studies revealed major differences in the potential steady state and dynamic properties of the different regulatory mechanisms, and also in the metabolic cost associated with regulation.

In this work we compare the dynamic behavior of four fundamentally different regulatory mechanisms embedded in a gene regulatory cascade, in an isogenic background. We constructed an *E. coli* strain in which production of the LacI repressor can be regulated both at the level of transcription initiation and translation initiation, and, in addition, the activity and stability of the protein can be regulated allosterically and by proteolytic degradation. The dynamics of the four different mechanisms of regulation are studied experimentally and analyzed using a mathematical model.

## Results

### Construction of a synthetic gene regulatory cascade

**Figure 1:**
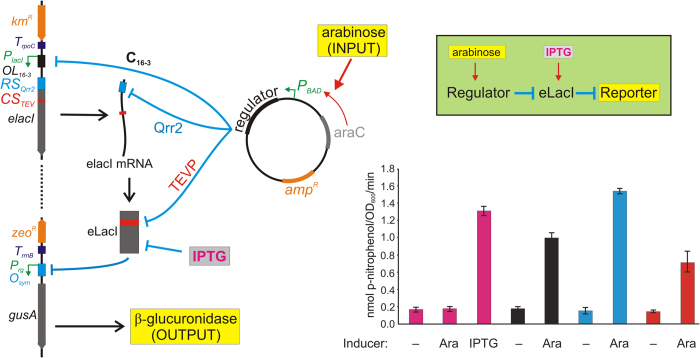
Structure and function of the engineered regulatory cascade. The logic structure of the cascade is shown in the green box. The left panel shows the elements and interactions of the system. See the *Methods* section for the details of construction. *Km*^*R*^, kanamycin resistance cassette; *T*_*rpoC*_, *rpoC* terminator; *P*_*lacI*_, LacI promoter; *OL*_*16-3*_, left operator for 16-3 C repressor; RS_Qrr2_, recognition site for Qrr2 binding; CS_TEV_, coding sequence for TEVP recognition site; eLacI, engineered LacI; *zeo*^*R*^, zeocin resistance cassette; *T*_*rrnB*_, *rrnB* terminator; *P*_*rg*_, modified lacUV5 promoter in front of the reporter gene; *O*_*sym*_, symmetric LacI operator sequence; *gusA*, *uidA* gene; *amp*^*R*^, ampicillin resistance cassette; *P*_*BAD*_, *P*_*BAD*_ promoter. The right panel shows the reporter gene activities obtained in the presence and absence of the four regulators. Exponentially growing cultures of SAH317 carrying pBAD24 (vector control, magenta bars), pBAD-C_16-3_ (black bars), pBAD-qrr2 (blue bars), and pBAD-TEVP (red bars) were induced with 0.2% arabinose (Ara) or 100 μM IPTG as indicated, and the cells were harvested after an additional 4 hours of growth. β-gluc activities are shown as the rate of PNPG conversion. In the absence of regulators, eLacI (engineered LacI containing the TEVP recognition sequence) is produced and represses *gusA* transcription. In the presence of either one of the four regulators, e*lacI* is negatively regulated by one of four shown mechanisms, resulting in production of *gusA*.

A synthetic GRC was designed in *E. coli* MG1655 (Fig. 1, green box). The transcriptional repressor of the Lac operon, LacI, was chosen as the target for regulation by four different mechanisms. To establish a readout for the system, the promoter of the *uidABC* operon was replaced by a LacI-controlled promoter^12^. Expression of the reporter protein β-glucoronidase (β-gluc, encoded by *uidA*), which inversely correlated with LacI activity, was quantified by measuring the level of β-glucuronidase activity in cell extracts (Fig. 1). We used a long-lived reporter because most *E. coli* proteins have half-lives much longer than the doubling time of cells^13^.

The chromosomal *lacI* gene was engineered to put LacI expression under the control of a transcriptional repressor, a small RNA (sRNA), and a protease, while preserving allosteric control by the synthetic lactose analogue isopropyl-thio-β-D-galactoside (IPTG). The chosen regulators all originate from organisms other than *E. coli* to ensure that they do not have specific endogenous targets, other than the engineered LacI (eLacI). Transcriptional repression was achieved by placing a promoter in front of *lacI*, which overlaps with an operator recognized by the C repressor protein of bacteriophage *16-3*^14^. To regulate LacI mRNA stability and translation, a DNA fragment encoding a binding sequence for the *V. cholerae* quorum regulatory RNA 2 (Qrr2) was placed between the transcription start site and the LacI start codon. A protease recognition sequence was inserted into the LacI protein by site-directed mutagenesis of the *lacI* coding sequence, placing LacI protein stability under the control of the tobacco etch virus protease (TEVP). Finally, IPTG was used as an allosteric inhibitor of LacI protein activity. Strong transcriptional terminator sequences were placed in front of both the *gus* reporter gene and the e*lacI* gene, ensuring that no run-through transcription from upstream genes would interfere with the system.

The genes encoding the *16-3* C repressor, the Qrr2 sRNA, and TEVP were inserted separately into plasmid pBAD24^15^, under the control of the arabinose-inducible *P*_*BAD*_ promoter. Therefore, in a strain carrying a regulator-coding pBAD24 derivative, expression of the regulator could be induced by addition of arabinose to the growth medium. Because this study aims to explore system dynamics, we chose to study *ara*^+^ strains, in which the *P*_*BAD*_ promoter of the pBAD24 plasmid responds much faster to depletion of arabinose than in ∆*araBAD* strains^15^.

### Induction of reporter gene expression

The synthetic GRC is designed to exhibit two stable states, ON and OFF. In the presence of any of the regulators, eLacI is repressed and, as a consequence, the *gusA* reporter gene is transcribed to produce high β-gluc levels (ON state). In the absence of the regulators, eLacI is produced and active. As a consequence, it represses the reporter gene resulting in low levels of β-gluc (OFF state) (Fig. 1).

The proper behavior of the constructed GRC was confirmed by comparing the β-gluc activities in the presence and absence of inducers for each regulatory mechanism, as shown in Fig. 1, bottom right panel. In the absence of induction, β-gluc was produced at a low basal level, indicating high eLacI activity in the cells (Fig. 1, first bar). When cells were grown in the presence of IPTG, they displayed a high level of β-gluc activity, due to the inactivation of eLacI by IPTG (third bar). The same was observed when the *16-3* C repressor (black bars), Qrr2 sRNA (blue bars), or TEVP (red bars) were induced by addition of arabinose. Thus each regulator is able to regulate eLacI. Arabinose did not affect the β-gluc activity in cells carrying the pBAD24 vector (second bar). We used 0.2% arabinose because at this concentration, transcription from the *P*_*BAD*_ promoter is expected to be induced in the entire cell population^16^.

### Regulator effects on the dynamics of the experimental system

**Figure 2:**
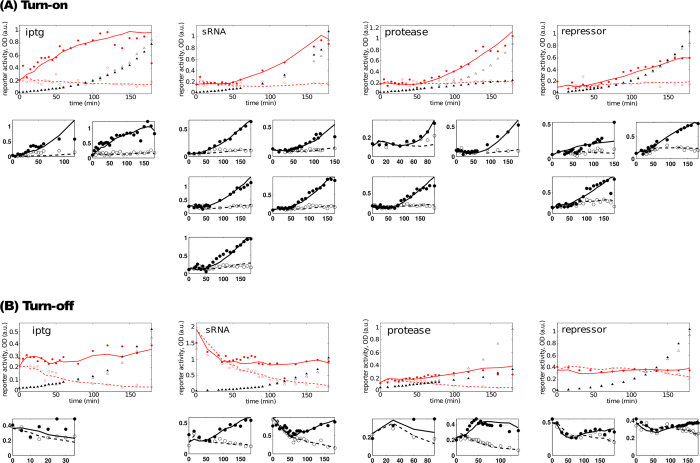
Fits of the mathematical model to the dynamical experimental data. Filled and open circles show dynamics of reporter activity upon inducer addition and removal in SAH317 cells containing one of the four plasmids (IPTG: pBAD24, repressor: pBAD-C_16-3_, sRNA: pBAD-qrr2, protease: pBAD-TEVP). Solid and dashed lines in each panel show the fit to the theoretical model. Growth curves in the presence (▲) and absence (∆) of the inducer are also shown for the experiments in large panels. (**A**) Turn-on experiments: Cultures were grown in the absence of the inducer molecule for minimum 10 generations. At time 0, the cultures were split in two and the inducer (0.2% arabinose or 100 μM IPTG) was added to one half (solid circles), while the other half continued growing in the absence of the signal molecule (open circles). Reporter β-gluc activity is shown as the rate of PNPG conversion (a.u.). (**B**) Turn-off experiments: Cultures were grown for minimum 10 generations in the presence of the inducer molecule (0.2% arabinose or 100 μM IPTG). Cultures were then split in two, and extracellular inducer molecules were removed from one half by centrifugation, washing, and resuspension of the cells in fresh medium (open circles). The other half of the culture was placed on ice in the interim time (solid circles). At t = 0, the cultures were returned to 37 °C to resume growth. The values of the fitted parameters that are common to all experiments are: 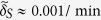, 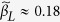, 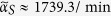, 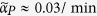, 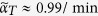, 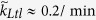, 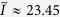, and *h*_*T*_ ≈ 1.016. In addition, the parameters from the steady-state IPTG fit, 
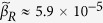
 and *h*_*L*_ ≈ 1.2, were also used to generate the theoretical fits (see Fig. 3). The mRNA half-lives were kept fixed at 4 minutes, based on our experimental measurements, i.e., *γ*_*Rm*_ ≈ 0.173/min, *γ*_*Lm*_ ≈ 0.173/min. The fitted parameter values for 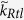 and the levels of the mRNAs and proteins at time zero, which were different for each experiment, are listed in Supplementary information Table S1.

To examine the response of the systems to appearance of the inducer molecule (OFF → ON transition), exponentially growing cell cultures containing the synthetic GRC were split in two, and saturating levels of arabinose (or IPTG in the case of small molecule regulation) were added to one of the two cultures to induce production of the regulator. Samples were collected at regular time intervals and β-gluc levels were determined. The large panels in Fig. 2A shows a representative example of these experiments for each regulator. As expected, β-gluc activity was initially low in all four cases, and remained low in the control cultures that did not receive the inducer (open circles). However, in the presence of inducer, cells switched to the ON state with different dynamics depending on the regulator.

After addition of IPTG the intracellular β-gluc level started to increase instantly. By contrast, significant accumulation of β-gluc occurs only after about 40 to 60 minutes (1 to 2 cell generations) in the case of the macromolecular regulators. In three of the four cases, induction did not affect cell growth (Fig. 2A, triangles), however, TEVP expression impaired growth temporarily. After a couple of hours, the TEVP-expressing culture resumed growth at the same rate as the other cultures. Examination of induced cells by microscopy revealed that while the majority appeared normal, some of the protease-expressing cells were elongated (data not shown).

We next examined how quickly the effect of inducer removal is transduced through the GRCs. The large panels in Fig. 2B shows a representative example of transition from a regime where eLacI is repressed by one of the regulatory mechanisms to a regime where eLacI is derepressed (ON → OFF transition). An exponentially growing culture containing saturating levels of the inducer was split in two, and the inducer was removed from one of them by centrifugation and washing with fresh medium (open circles), while the other half continued growth in the presence of the inducer (closed circles). The response speed varied greatly between systems employing the four regulators. We found that the removal of the inducer molecule was almost immediately reflected in the β-gluc activity in the case of IPTG, followed by the protease, the sRNA, and eventually the transcriptional regulator after about 140 min.

### A mathematical model reproduces the experimental data

Figure 2 shows that the dynamic behavior of the GRCs is indeed affected by the regulatory mechanism employed to regulate eLacI. However, our experimental system could not reveal all the possible features of the GRCs because the choice of the regulatory elements constrains crucial parameters, such as parameters related to the physical interaction between the regulators and their targets (binding strength) and by the intracellular concentration of the regulators. Because engineering the experimental system to change these parameters is difficult and time consuming, we developed a mathematical model, based on the known biological mechanisms of action of the molecular components that make up the synthetic GRC, which allows simulations of the system starting with pre-defined conditions and parameters. The theoretical framework and the model equations are described in *Methods*. We used differential equations to model the dynamic changes in the concentrations of system components, with standard formalism to model different interactions: the Hill equation to model transcriptional activation and repression, and the law of mass action to model protein-protease interaction, protein-inducer interaction, and sRNA-mRNA binding^17^. We used the model equations to simulate each of the induction and inducer removal experiments. All dynamic experiments and the associated fits are shown in Fig. 2. This fit shows only that standard formalisms used to model transcription factor, sRNA, proteolytic and allosteric regulation are *sufficient* to model our synthetic system. We have not attempted to demonstrate *necessity* of the interactions and specific mathematical terms included in our model. Nor have we attempted to find the best possible fit, or determine fitted parameter values with high accuracy, because our intent is to explore a wide range of parameter values to determine the dynamical capabilities of a system similar to our synthetic system. The chosen parameter set has biologically reasonable values for Hill coefficients, regulator production rates, and life-times of key components, and the model captures all our biological knowledge about the individual parts and interactions in the synthetic GRC. Therefore, in our view, this model is an appropriate starting point for theoretical analysis.

The life-times of the e*lacI* and reporter mRNAs were fixed in all the fits to be 4 minutes, based on experimental measurements of the e*lacI* mRNA half-life, which gave a value of 4-5 minutes (see *Methods*).

**Figure 3:**
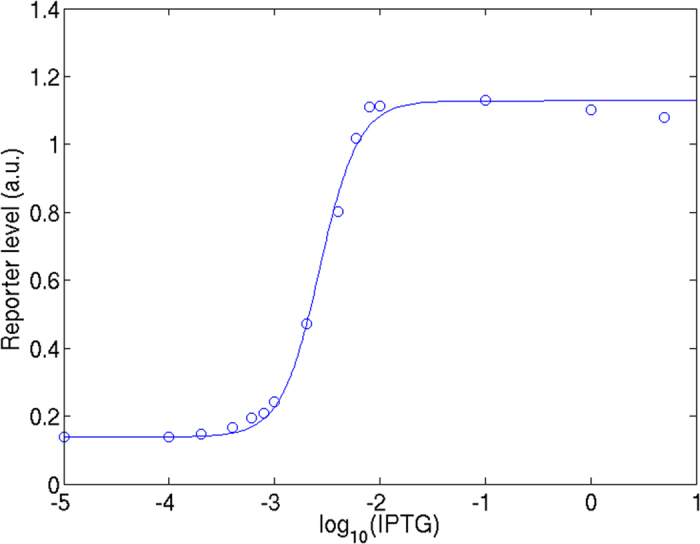
Fits of the mathematical model to steady-state experimental data. Circles show the reporter level 4 hours after addition of IPTG, as a function of IPTG concentration (mM). Solid lines show the plot of *R* (*steady-state*) vs. *I*, from eq. 23. The parameters of this fit were: *R*_*max*_ ≈ 1.96, 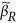
≈5.9 × 10^−5^, 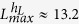, *β*_*I*_ ≈ 0.09, *K*_*I*_ ≈ 0.001, *h*_*I*_ ≈ 2.3, and *h*_*L*_ ≈ 1.2.

The parameters, 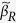 and *h*_*L*_ were chosen from separate fits to the steady-state β-gluc levels of the reporter strain SAH540 grown in the presence of different concentrations of the IPTG inducer (Fig. 3). We did not use similar steady-state data from the plasmid-encoded regulators because the response of the pBAD promoter to the arabinose inducer is all-or-none, that is, sampling a range of arabinose concentrations does not explore a range of regulator production rates—instead, it changes the ratio of ‘off‘ and ‘on‘ cells (ones that produce the regulator at low and high levels, respectively)^16^.

### Dynamics of the regulators and eLacI inferred from the model

**Figure 4:**
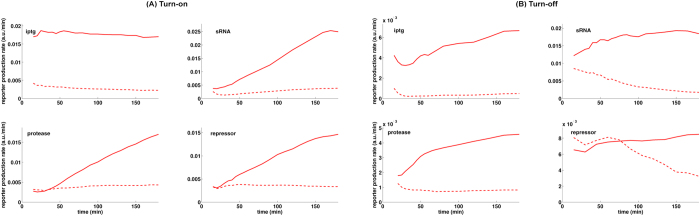
Reporter production rates determined from the simulated turn-on (A) and turn-off (B) experiments shown in Fig. 2. Solid red curves are the reporter production rates for the fits to the experimental cultures grown in the presence of arabinose, and dashed lines are for the fits to the experimental cultures grown without arabinose.

The eLacI protein exerts its effect on the reporter only at the level of production. However, in our experimental set-up the observed changes in reporter protein levels reflect both production and dilution due to cell growth (see Supplementary Information Figure S1). Because cell growth was recorded in the experiments, the effects of these processes on reporter protein levels can be computed separately. Fig. 4A, B show simulations of the reporter production rates in response to the addition or removal of arabinose, respectively. Since the reporter mRNA has a short half-life, the eLacI level can be inferred quite accurately from the reporter production rate (i.e., when the production rate is 0.025 per min, half of its maximum value of 0.05 per min, it means the LacI level is very close to that required for half-repression of the reporter promoter). Because addition of IPTG leads to immediate inactivation of eLacI, the delay in obtaining maximal reporter levels consists almost exclusively of the time required for the reporter protein to accumulate. By contrast, the three GRCs containing different macromolecular regulators show a delay in reaching the maximal reporter production rate, which is composed of the time required to produce the regulators, and the time the regulators need to exert their effect on the concentration of active eLacI. After an initial lag of about 25 minutes the reporter production rate keeps increasing for more than 4 cell generation times (Fig. 4A, solid lines).

Previous reports predicted that sRNA-mediated regulation has a faster response time than repressor-mediated regulation^8^,^11^ because sRNAs inhibit translation of existing target mRNAs, whereas transcriptional regulation prevents the production of new mRNAs but does not prevent translation of existing target mRNAs. However, this difference was not reflected in the turn-on dynamics of GRCs containing the Qrr2 sRNA and the 16-3 C repressor. The explanation is that the difference between the repressor and sRNA-mediated response speeds is small compared to the protein production and degradation times, which are the major determinants of the response speed in these GRCs, since the e*lacI* mRNA is produced at a low rate and its half-life is short.

Figure 4B shows that removal of IPTG leads to immediate repression of reporter transcription (dashed line). Thus, the measured reporter activity in this case (Fig. 2B) represents pre-existing reporter protein that has not yet been diluted out due to cell division. Fig. 4B shows that upon removal of arabinose, the repressor maintains eLacI inhibition for about 2 hours (~4 cell generations), while repression of eLacI is removed after one cell generation in the case of proteolytic degradation and sRNA regulation.

### The effect of regulator production rate on steady state reporter levels

The actual eLacI level depends on the number of regulator molecules relative to their binding affinities. To explore whether repression by the three macromolecular regulators could be improved by increasing their intracellular concentration, we calculated the steady state reporter levels as a function of regulator production rates.

**Figure 5:**
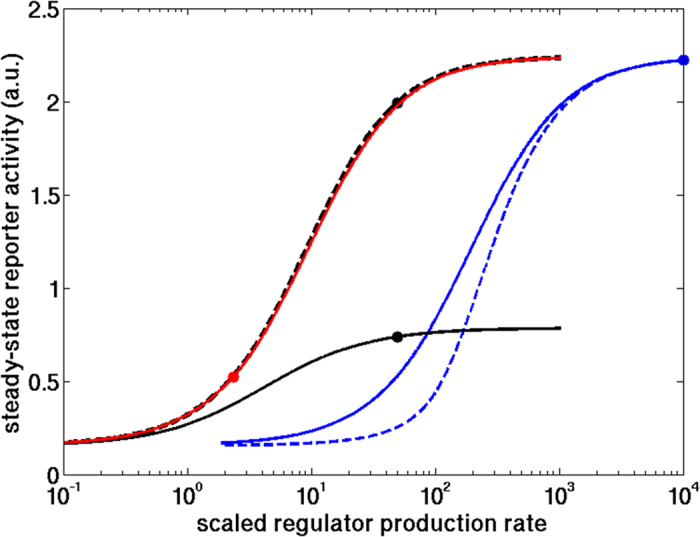
Simulated steady state reporter levels as a function of scaled (i.e., normalized) regulator production rate. Circles indicate the parameters found from fits to the experimental dynamic data. The solid red, blue, and black curves show simulations with the protease, the sRNA, and the repressor, respectively. The dashed black line indicates simulation with the repressor without a leak term, and the dashed blue line a sRNA with 100-fold increased sRNA-mRNA binding rate. The steady-state reporter level is expressed in arbitrary units (a.u.). The regulator production rate is scaled as follows: (i) for repressor, the rate is divided by the dissociation constant of its binding to its operator and by the cell division rate, (ii) for sRNA, the rate is divided by the maximal production rate of LacI mRNA, (iii) for protease, the rate is multiplied by the LacI-protease association rate and divided by the square of the cell division rate (see Supplementary Information “Interpreting the rescaled parameters”).

Figure 5 shows that the production rate of the protease in our simulations is in a regime where the steady state reporter level is very sensitive to changes in regulator production rate (red circle). Thus, protease-mediated regulation can be improved substantially simply by increasing the regulator production rates. By contrast, the sRNA and transcriptional repressor are produced at rates close to saturation and thus cannot be improved much by increasing the production rate (blue and black circles, respectively).

To properly reproduce the experimental data, the model equation for repressor regulation contains a small leak term (*β*_*L*_) that accounts for residual transcription even in the presence of saturating amounts of repressor. This residual transcription is responsible for the inefficient repression of eLacI by the repressor. As seen in Fig. 5, removal of the leak term (*β*_*L*_ = 0, dashed line) allows repressor-mediated regulation of eLacI to reach the same theoretical maximum efficiency as sRNA- or protease-mediated regulation. Thus, the observed relative weakness of repressor-mediated regulation compared to the other regulatory mechanisms results from the specific interaction of the 16-3 C repressor with its target DNA site and RNA polymerase, and is not due to the regulatory mechanism *per se*.

Figure 5 also contains information on the sensitivity of the systems to small perturbations in regulator production rates. Although regulation by sRNAs has been predicted to be ultrasensitive^8^,^11^,^18^, we observed similar graded responses for the three regulators. However, ultrasensitive sRNA regulation could be produced in the model by increasing the efficiency of sRNA-mRNA pairing (Fig. 5, dashed blue line).

### Potential dynamical properties of regulatory cascades

As mentioned before, certain parameters are constrained by the specific molecular identity of the regulators used in the experimental system but can be changed individually in the mathematical model. Therefore, in this section we explore a wider range of parameter values in *in silico* simulations to understand the potential response and recovery dynamics that can be obtained using each different type of regulator and to determine which aspects of the observed dynamics are due to inherent differences between the regulatory mechanisms. Because we are specifically interested in the maximal possible inhibitory effect of each regulator on eLacI, we focus on those parameters that can improve the inhibitory action of each regulator.

**Figure 6:**
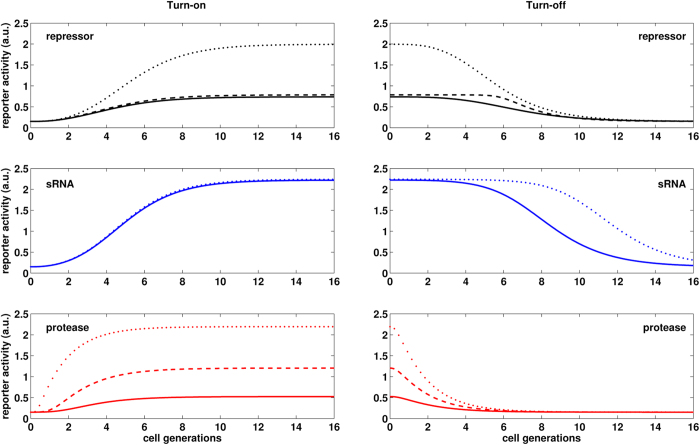
Simulations of system dynamics after inducer addition and removal. Left panels show simulations of induction experiments for each of the three regulators, while the right panels show simulations of inducer removal. Solid curves represent simulations performed with the parameter set determined by fitting the model to the experimental measurements. Dashed and dotted lines show simulations with alterations of regulator parameters. Black dots: repressor with no residual promoter activity (*β*_*L*_ = 0), black dashes: repressor with higher operator binding cooperativity (*h*_*L*_ = 4), blue dots: higher rate of sRNA production (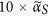), blue dashes (overlaps with the solid line): higher rate of mRNA binding by the sRNA (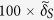), red dots and dashes: higher protease production rates (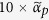 and 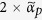, respectively).

The eLacI level in the repressor-controlled GRC depends on (i) the level of the repressor relative to its binding affinity, (ii) the cooperativity of operator binding, (iii) the residual activity of the repressor-bound promoter, and (iv) the degradation/dilution rate of the repressor. As shown in Fig. 5, repression of eLacI cannot be improved by further increasing repressor production. Therefore we simulated the effects of increased operator binding cooperativity, and decreased residual promoter activity on turn-on and turn-off dynamics (Fig. 6, upper panels). The turn-on response dynamics of the repressor controlled GRC are generally delayed and graded. The turn-off dynamics are also delayed, and the duration of the delay increases with increased operator binding cooperativity (dashed line). Non-leaky repression of transcription does not change the dynamics substantially but inactivates eLacI more efficiently, almost as strongly as IPTG (dotted lines).

The response dynamics of the sRNA-controlled GRC depends mainly on two parameters, the production rate of sRNA relative to the production rate of its target, and the rate of sRNA-mRNA pairing^10^. Therefore we investigated the dynamic behavior of GRCs which either contains a sRNA regulator that pairs more efficiently with its target mRNA or a sRNA which has an increased maximal production rate (Fig. 6, middle panels). The sRNA-controlled GRC shows delayed and graded turn-on dynamics regardless of the parameter choice. This is because eLacI is stable, and although the sRNA shuts down further eLacI production, the dynamics are governed by the dilution rate of the existing eLacI proteins. The turn-off response of the sRNA-controlled GRC is substantially delayed and the duration of the delay increased when the production rate of the sRNA was increased (dotted line).

In the experimentally constructed protease-controlled GRC the turn-on response was similar to what was observed in the other two GRCs, i.e. it showed a graded response after an initial delay (Fig. 6, bottom left panel, solid line). However, in this system the protease is produced at a rate far below saturation (Fig. 5). Therefore we examined the effect of increasing the protease production rate (Fig. 6, bottom left panel, dashed and dotted lines). Increasing the protease production rate substantially changed the turn-on dynamics of the system, resulting in rapid signal transduction through the GRC. We note that the scaled parameter for the protease production rate (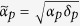) includes the parameters for target recognition and inactivation rates as well.

## Discussion

In this work we constructed a gene regulatory cascade in which a transcriptional repressor can be regulated both at the level of transcription initiation and translation initiation, and, in addition, the activity and stability of the protein can be regulated allosterically and by proteolytic degradation. Using this synthetic regulatory system we gained a quantitative insight into how the molecular mechanisms underlying regulation affect signal propagation through GRCs.

Based on previous mathematical descriptions of regulatory interactions we constructed a mathematical model which could reproduce the experimental measurements.

### Response dynamics of gene regulatory cascades

In principle, four fundamentally different responses could be produced with the GRCs. These responses differ in the duration of the delay of the response of reporter protein level after the change in the input signal, and in the transition between the ON and OFF states (sharpness of the response). We define the duration of the response delay as the time needed to reach the dynamic response period of the system. The dynamic response period is the time interval where the reporter level changes significantly over time, i.e. its value is above the minimal level by 10 to 90% of the total dynamic range. The sharpness of the response can therefore be defined as the time span between entering and leaving the dynamic response period.

The dynamics that the GRC could perform in the simulations are summarized in Table 1. The delay in the turn-on response is an inherent nature of the sRNA- and repressor-controlled GRCs but can be engineered in the protease-controlled system (Table 1). However, because all three regulators have long half-lives, the delay in the turn-off response depends largely on the amount of excess regulator molecules in the induced state of the systems. The delay thus corresponds to the time required for the clearance of excess regulators from the cell. Therefore, our simulations indicate that the duration of the time delay is not a function of the regulatory mechanism *per se*, and it can be engineered in all three cases by altering the maximal regulator production rate. Because the reporter protein is stable, the sharpness of the turn-off dynamics is limited by the dilution rate of the reporter protein. Therefore, even though typical repressors such as the 16-3 C repressor produce a graded response and efficient proteolytic regulation result in a sharp response^19^, these features are not fully manifested at the level of the reporter dynamics.

Direct allosteric regulation of eLacI by IPTG was the only mechanism that resulted in substantial expression of the reporter protein within the first cell generation time in our experimental system. However, simulations show that a similar fast response can be achieved by an efficiently produced protease. Because the number of eLacI molecules is relatively low in the system, and the protease acts catalytically, our simulations indicate that the response speed of the protease-regulated GRC is mostly determined by the time scale of reporter production and accumulation, similar to allosteric regulation. In the case of transcriptional- and sRNA-mediated repression, the time needed for dilution of existing eLacI proteins adds a substantial delay to the response time. These results are in agreement with the observation that in *E. coli*, a free living organism, which needs to respond quickly to external stimuli, at least 50% of the transcription factors are regulated allosterically by small molecule signals^20^, and the average path length in its transcriptional network is only 1.36 steps, i.e. most transcription factors regulate their targets directly^21^. However, the time delay introduced by the two-step GRCs involving transcriptional regulators or sRNAs can be useful in the design of regulatory circuits performing more complex dynamics^22^,^23^.

### Evolutionary aspects

The specific advantages of the regulatory mechanisms employed here have been studied extensively. However, these regulatory mechanisms are often parts of larger regulatory architectures such as the GRC studied in this work. This raises the question whether the unique features of the regulatory mechanisms are manifested in the overall function of a larger regulatory cascade. That is, whether acquisition of new regulatory links in cellular networks are governed by selective pressure for properties that are restricted to a particular type of regulator. In our experimental system, the chosen regulators all originated from organisms other than *E. coli*, therefore the system mimics addition of new regulatory links to the cellular network by horizontal gene transfer. Similar to our experimental system, regulatory links acquired by horizontal gene transfer are functional but not optimized for overall network performance.

Our results suggest that in some cases there may be no selection pressure for the mechanism of regulation in the evolution of regulatory links in networks. For example, the experimental observations reported here directly show that the second step of a GRC can completely mask dynamical differences caused by different molecular mechanisms regulating the first step of the cascade. Our model simulations can be used to dissect this further: sRNA action can be ultrasensitive, but the sensitivity of the two-step GRC examined here was dominated by the Hill coefficient of LacI repression in the second step (Fig. 5). Similarly, although a protease can cause LacI levels to decrease faster than a transcriptional repressor can, the time required for the reporter level to rise in response to an induction signal will be similar for both protease- and repressor-mediated regulation if the LacI production rate is sufficiently high (Fig. 4).

In such cases, the absence of selection pressure for properties of a particular regulatory mechanism means that the choice of regulatory mechanism will be governed by other factors such as the availability or evolvability of existing regulators, and parameter constraints imposed by existing interactions between the regulator and other targets or between the target and other regulators.

In other cases, the same regulatory mechanism appears in a given genetic context within different GRC’s and in different organisms. We may speculate about the selective advantage of such a specific regulatory feature. For example, genes that are positively regulated by an activator are often negatively regulated by a transcriptional repressor (as opposed to other types of negative regulators)^24^. In such cases, we would argue that transcriptional repression is preferred because the production rate of target mRNA and protein depends on the level of the transcriptional activator, and therefore the number of target molecules changes for sRNA (which targets mRNA) and proteolytic control (which targets the protein product) but remains the same for repressor mediated control (which targets DNA). That is, repressors can inhibit transcription activation over a wide range of activator levels^25^, while the efficiency of the sRNA and proteolytic systems may decrease substantially with increasing activator levels^26^,^27^. This argument is further supported by the outcome of simulations of the GRC constructed here with increased rates of e*lacI* transcription (data not shown). We note that this particular disadvantage of the post-transcriptional regulatory mechanisms can be overcome by co-regulation of the regulator and target promoters, so that regulator production increases concurrently with an increase in target production. An example of such co-regulation can be seen in the iron control system of *E. coli* where the Fur protein controls transcription of both *sodA* and the small regulatory RNA RyhB, which inhibits translation of the *sodA* mRNA^28^.

## Methods

### Bacterial strains and culture conditions

The bacterial strains and plasmids used in this study are listed in Supplementary Information Table S2. All strains were grown aerobically at 37 °C in yeast tryptone (YT) medium (0.8% tryptone, 0.5% yeast extract, 0.5% NaCl). Concentration of antibiotics used was as follows: chloramphenicol 25 μg/ml, zeocin 80 μg/ml, ampicillin, rifampicin, and kanamycin 100 μg/ml.

### Plasmid and strain construction

The GRC was created in *E. coli* MG1655^29^ by engineering the chromosomal regions containing the regulatory gene *lacI* and the reporter gene *uidA (gusA)* (SAH317)*. uidA* was placed under the control of the LacI-controlled cAMP-CRP-independent UV5 promoter, containing a symmetric *lac* operator^12^. The DNA encoding this promoter was synthesized using the partially complementary RIUV5up and UV5dnPst primers. The PCR product was digested using the restriction enzymes *Eco*RI and *Pst*I, and ligated into pSEM2027^30^. A PCR was performed on the resulting plasmid using the primers UidRzdn and gusseqdn^30^ and the synthesized DNA fragment was inserted upstream of the *uid*A gene of the *E. coli* MG1655 chromosome as described before^30^, generating SAH1.

The promoter and regulatory region of the *lacI* gene and the *lacI* gene itself were engineered to allow transcriptional, translational, and proteolytic regulation. To prevent read-through transcription from upstream promoters, the *rpoC* terminator was amplified from pSA850^31^ by PCR using the NsiRpoCTup and RpocTdnAcc primers. The resulting PCR product was digested by *Nsi*I and *Acc*65I and inserted between the same sites in pBBR1MCS2^32^. A synthetic promoter was constructed using the primers RIupPL and dnPLPst. This promoter carries the left operators of bacteriophage 16-3 and therefore it is repressed in the presence of the C repressor of the phage^33^. One position was randomized to create constructs with different promoter strengths. After testing the different versions, the promoter containing an A in the position of the variable nucleotide was chosen for insertion between the *Acc65*I and *Xba*I sites of the pBBR1MCS2 plasmid containing the *rpoC* terminator, generating pSAH21. The LacI coding region was amplified from plasmid pEVS141^34^ by PCR using primers HindIIILacIup and lacIdnAtXb. The resulting PCR product was digested with *Hind*III and *Aat*II and ligated into pSAH21 to generate pSAH32. The *Hind*III and *Apa*LI DNA fragment of pCR2.1-LacI-sRNA-32BS, encoding the leader sequence of *V. cholerae hapR*, which is a confirmed binding sequence for the quorum regulatory RNA 2 (Qrr2)^18^,^35^, was inserted between the operator elements and the LacI coding sequence of pSAH32, to generate pSAH44. To insert the TEV protease cleavage site, the LacI coding sequence in pSAH44 was altered by Quick change mutagenesis (QuickChange II Site-Directed Mutagenesis Kit, Alignment Technologies) using primers qcTEV100up and qcTEV100dn, generating pSAH47. The engineered LacI (eLacI) protein encoded by pSAH47 carries a TEV protease cleavage site from amino acid position 100 to position 106. This region was reported to be tolerant to amino acid substitutions^36^. The engineered *lacI* (e*lacI*) gene together with the upstream rpoC terminator and control region and with the downstream Km^R^ gene of pBBRMCS2 was amplified by PCR from pSAH47 using primers LacIuprecomb and LacInydnrecomb and transferred to the chromosome of SAH1 by recombineering^37^, generating strain SAH317.

The *araDABC* genes in SAH317 were replaced with a chloramphenicol resistance cassette by recombineering^37^, generating SAH538. The cassette was amplified from pKD3 using primers ARACDELREV and ARADDELFWD.

Genes encoding the three regulators that were used to control LacI activity were cloned separately into plasmid pBAD24^15^, downstream of the arabinose controlled *P*_*BAD*_ promoter. The gene encoding repressor C of bacteriophage 16-3^33^ (GenBank: NC_011103.1) was amplified using primers pBAD24C16-3up and pBAD24C16-3dn and inserted between the *Xba*I and *Hin*dIII sites of pBAD24, generating pBAD-C_16-3_. The gene encoding TEVP was cloned into pBAD24 by digesting the plasmid pTH24:TEVsh^38^ by *Xba*I and *Pvu*I and digesting pBAD24 using *Nhe*I and *Pvu*I, generating pBAD-TEVP. The *qrr2* expression plasmid was constructed by digesting pCR2.1-qrr2 (Eurofins MWG gene synthesis), with *Bam*HI and *Hind*III and cloning it into pBAD24 using *Bam*HI and *Hind*III, generating pBAD-qrr2. Sequences of all the constructs were verified (Eurofins MWG operon). The details of the construction are shown schematically in Fig. 1. The oligonucleotides used are reported in Supplementary Information Table S3.

### Assay of β-glucuronidase activity

For the endpoint measurements shown in Figs 1 and 3, overnight cultures were diluted in YT medium containing 100 μg/ml ampicillin to an OD_600_ = 0.001 and grown at 37 °C. Inducer was added at OD_600_ = 0.02 when present. Samples were collected after 4 hours. Volumes were adjusted to correspond to 1 mL of cells at OD_600_ = 0.65. Cells were collected by centrifugation and stored at −80 °C.

For studying system dynamics upon induction (Fig. 2, Turn-on), overnight cultures were diluted to an OD_600_ = 0.001 in YT medium containing the appropriate antibiotics. Inducer was added at OD_600_ = 0.025 when present. Samples were collected at different time points as described above.

For studying system dynamics upon inducer removal (Fig. 2, Turn-off), overnight cultures grown in the presence of inducer were diluted to an OD_600_ = 0.001 in YT with the appropriate antibiotics and inducer. At OD_600_ = 0.025 cells were washed twice in YT to remove the inducer and resuspended in YT with the appropriate antibiotics for further growth. Samples were collected at different time points as described above.

β-glucuronidase (β-gluc) activity of the collected cells was determined as described previously^39^ with the modification that 0.5 mg/ml lysozyme was added to the cell suspension. The rate of reaction was extracted from the data by plotting a graph of OD_405_ versus time in minutes. The slope (S) of the curve was calculated and used to calculate the rate of reaction in nanomoles product produced per minute per OD_600_ using the formula (S/(0.018*OD_600_)) derived from^40^. Values represent the mean of each assay carried out in duplicate.

### Determination of elacI mRNA half-life

Exponentially grown cultures of SAH317 were treated with rifampicin to stop transcription, and total RNA was harvested from samples taken before addition of rifampicin and at 5, 10, 15, and 30 minutes after antibiotic addition using the RNeasy midi kit (Qiagen, Inc.,Valencia, CA, USA). First strand cDNA synthesis was performed using RevertAid H minus first strand cDNA synthesis kit (Thermo Fisher Scientific Inc.). Quantitative PCR reactions were conducted using primers LACIUPQPCR and LACIDNQPCR and the SsoAdvanced SYBR Green Supermix (BioRad, Hercules, CA, USA). Real-time PCR amplifications were performed using the BioRad CFX96 Real-Time PCR system.

### Development of the mathematical model

We model the concentration of the reporter, R, using the following two equations:


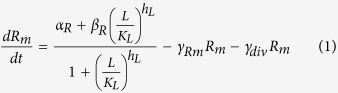



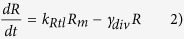


Here, *R*_*m*_ is the concentration of the mRNA transcript produced from the reporter gene, and *L* is the concentration of free LacI tetramers that are available to repress transcription of the reporter. The repression is represented by the cooperative Michaelis-Menten or Hill type term in eq. 1, with *K*_*L*_ being the dissociation constant of a LacI tetramer to its operator, and *h*_*L*_ the cooperativity of the binding, assuming that binding and unbinding of LacI to its operator site occurs at much faster timescales than transcription, translation, and degradation (the same type of assumption that is employed in Michaelis-Menten enzyme kinetics^17^). *α*_*R*_ is the maximal rate of transcription of the reporter gene (in the absence of LacI), and *β*_*R*_ is the rate of transcription when saturating levels of LacI are present. The reporter protein is stable, so its concentration only decreases due to dilution by cell division, at a rate *γ*_*div*_, whereas the mRNA, which is translated at a rate *k*_*Rtl*_, is degraded at a rate *γ*_*Rm*_ in addition to being diluted by cell division. The level of LacI, which is regulated in different ways, is modeled as follows:


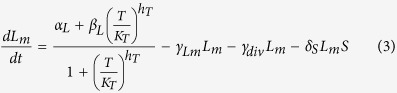



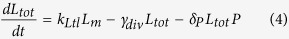



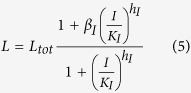


Here, *L*_*m*_ is the concentration of LacI mRNA and *L*_*tot*_ is the total concentration of LacI tetramers (both free and bound to IPTG, *I*). The LacI mRNA is translated at a rate of *k*_*Ltl*_and has a half-life of ln2/*γ*_*Lm*_. These equations assume that LacI protein has much longer half-life than the cell doubling time. Because binding and unbinding of IPTG to the LacI tetramer occurs at a much faster timescale than transcription, translation and degradation, the amount of free LacI tetramer can be modeled using a Hill type term with *K*_*I*_ being the dissociation constant of IPTG-LacI binding and *h*_*I*_ being the cooperativity. The Hill type term in eq. 3 models the repression of LacI by a transcriptional regulator, *T*, in the same way as the repression of the reporter by LacI in eq. 1, with the same assumptions about timescales. Eq. 3 also allows for a sRNA, *S*, that binds to and degrades (or irreversibly inactivates) LacI mRNA. Similarly, eq. 4 allows for a protease, *P*, which actively degrades the LacI protein.

To model the dynamics of *T*, *S*, and *P*, we use the following equations:


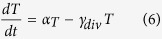



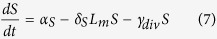



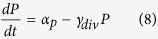


In eq. 6 we assume that the transcriptional regulator is stable and its concentration decreases only due to dilution by cell division. *α*_*T*_ is the effective production rate of T, which in our experiments depends on the presence of arabinose. Because our experiments involve either a fixed level of arabinose at all times, or switching between zero and a fixed level of arabinose, we have not explicitly modeled the concentration of the T mRNA. This neglects the possible transient dynamics of mRNA concentration when arabinose levels are altered, but this will not be a significant factor as long as the mRNA half-life is much shorter than the timescales of protein production and degradation.

In eqs 3 and 7, similar to previous models of sRNA regulation^8^,^11^,^18^,^41^, we assume that: (i) the degradation of the sRNA-mRNA complex is faster than the dissociation of the same complex, so that the binding is effectively irreversible; (ii) both the sRNA and the mRNA are inactivated upon complex formation^42^; (iii) translation of the mRNA is not possible after the complex with the sRNA is formed. Further, the half-life of the unpaired sRNA is long^43^ compared to when bound to the mRNA, so we have neglected that degradation term.

In eqs 4 and 8 assumptions about the protease are similar to those made for the other regulators: it is assumed to be stable and its binding to LacI is effectively irreversible, so only the term corresponding to formation of the LacI-protease complex is included. The protease molecules act catalytically to degrade eLacI.

The parameters in the above equations, except *α*_*R*_, are similar across all experiments. In addition, there are several more parameters related to the initial concentrations (at the time arabinose was added or removed from the system) of system components that can take different values in different experiments. *α*_*R*_ is allowed to take a different value for each experiment because it is an effective production rate of the reporter that combines the actual rate at which it is produced inside the cells, with the efficiency of cell permeabilization and subsequent color development in the β-glucuronidase assay, which may vary from day to day. However, the *α*_*R*_ value chosen for a particular experiment in the presence of arabinose is not allowed to be larger than the value for the corresponding experiment without arabinose—the logic for this is that *α*_*R*_ may at best be slightly reduced in the experiments with arabinose because the production machinery, such as RNA polymerases, might be occupied transcribing the regulators from the arabinose-induced promoters on multicopy plasmids.

### Fitting the mathematical model to the experimental data

We determined possible values for all the above parameters by using the model equations to simulate each experiment we have performed (multiple sets with each of the four regulators, with and without arabinose), and finding a parameter set that minimizes the square of the relative distance between the theoretical reporter concentration and the actual observed reporter value, summed over all time points and over all experiments (Fig. 2). This ‘least-square fit’ is determined by a stochastic gradient descent search of parameter space—random steps in parameter space are repeatedly attempted, and accepted only if they reduce the least-square distance, until the trajectory settles to a local minimum. This procedure is repeated starting with many different initial conditions to identify a sufficiently well-fitting local minimum. Fig. 2 shows the application of this algorithm to the full data set. Note that there may well be other models or other parameter sets that fit the data equally well or better. We only claim sufficiency of this model, which is demonstrated by the fit in Fig. 2. In Supplementary Information Figure S2, we show that fitting the model to a subset of the data produces a similar parameter set.

Because the experimental data consists only of reporter concentrations, and not concentrations of the components of the system, we could not determine all the parameters independently by fitting to data. The combinations of parameters that were independently determined from the fit were (see Supplementary Information):


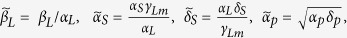
 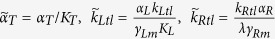



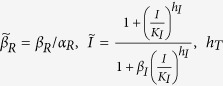


The dilution rate, γ_div_, was directly determined from the optical density measurements, and the half-lives of *eLacI* and reporter mRNA were fixed in all fits to be 4 minutes, based on our experimental determination of *eLacI* mRNA half-life (see *Methods*, above). In addition, all the initial condition parameters were determined. *α*_*R*_ values for each experiment also came out of the fit, but because reporter levels are known only up to an arbitrary multiplicative factor, these values have little biological meaning.

### Fitting parameters to steady-state IPTG experiments

The parameters, 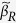 and *h*_*L*_ were, in fact, kept fixed in the fits to the dynamic data. The values chosen came from separate fits to the observed reporter levels from the steady-state experiments in the presence of different concentrations of IPTG shown (Fig. 3).

In steady-state, in the presence of IPTG but no other regulators of LacI, the dynamical equations are reduced to:


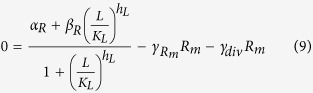



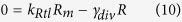



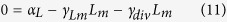



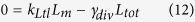



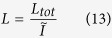


That is,


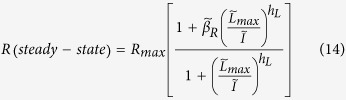


where


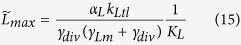


and


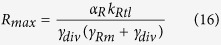


Thus, by least-square fitting the theoretical value for steady-state reporter as a function of IPTG concentration, from eq. 14, to our experimental data, we could determine the parameters *R*_*max*_, *L*_*max*_, *h*_L_, 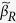, 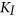, 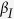, *h*_I_. Again, several different parameter sets fitted the data well, but we only found one set where *h*_*I *_≈ 2. We chose this set because IPTG is known to bind LacI tetramers at two independent sites, so we expect a cooperativity of close to 2^44^. The corresponding fit is shown in Fig. 3. Of these parameters, the ones directly usable in the fits to dynamic data were *h*_*L*_ and, *β*_*R*_/*α*_*R*_.

## Additional Information

**How to cite this article**: Hansen, S. *et al*. Effects of Four Different Regulatory Mechanisms on the Dynamics of Gene Regulatory Cascades. *Sci. Rep*. **5**, 12186; doi: 10.1038/srep12186 (2015).

## Supplementary Material

Supplementary Information

## Figures and Tables

**Table 1:** Potential response dynamics in the gene regulatory cascade. Response delays and speeds are expressed in units of cell generation time.

Regulator	Turn-on dynamics		Turn-off dynamics	
	Response delay	Response speed	Response delay	Response speed
Protease				
high production rate	0.5	3.4	0.5	3.6
low production rate	1.6	4.9	0.6	4.2
Repressor				
residual promoter activity	1.9	6.1	3.6	7.2
no residual promoter activity	2.5	6.4	3.0	6.4
high DNA binding cooperativity	1.8	6.1	6.0	4.4
sRNA				
low target binding rate	2.4	6.2	5.4	7.0
high target binding rate	2.4	6.2	5.4	7.0
higher production rate	2.4	6.1	8.6	7.1
