# Supplementary material for: Effects of Four Different Regulatory Mechanisms on the Dynamics of Gene Regulatory Cascades
Source: Sci Rep. 2015 Jul 17;5:12186. doi: 10.1038/srep12186 (PMC4505322; doi:10.1038/srep12186)
Supplement: Supplementary Information [file srep12186-s1.pdf]

**Supplementary Methods for**

# Effects of Four Different Regulatory Mechanisms on the Dynamics of Gene Regulatory Cascades

**by Sabine Hansen, Sandeep Krishna, Szabolcs Semsey, and Sine Lo Svenningsen**

## Contribution of production vs. dilution to reporter levels

The reporter level is governed by equation (2) in the main text:

$$\frac{dR}{dt} = kR_m - \gamma_{div} R$$

As mentioned in the main text, observed changes in the reporter level reflect both production (first term on the right hand side, above) and dilution due to cell growth (second term on the right hand side, above). Figure S1 shows the net production rate ($dR/dt$) and the dilution rate ($\gamma_{div}R$), as estimated from observed reporter levels and OD values in the eight experiments shown in the large panels in Figure 2 of the main text.

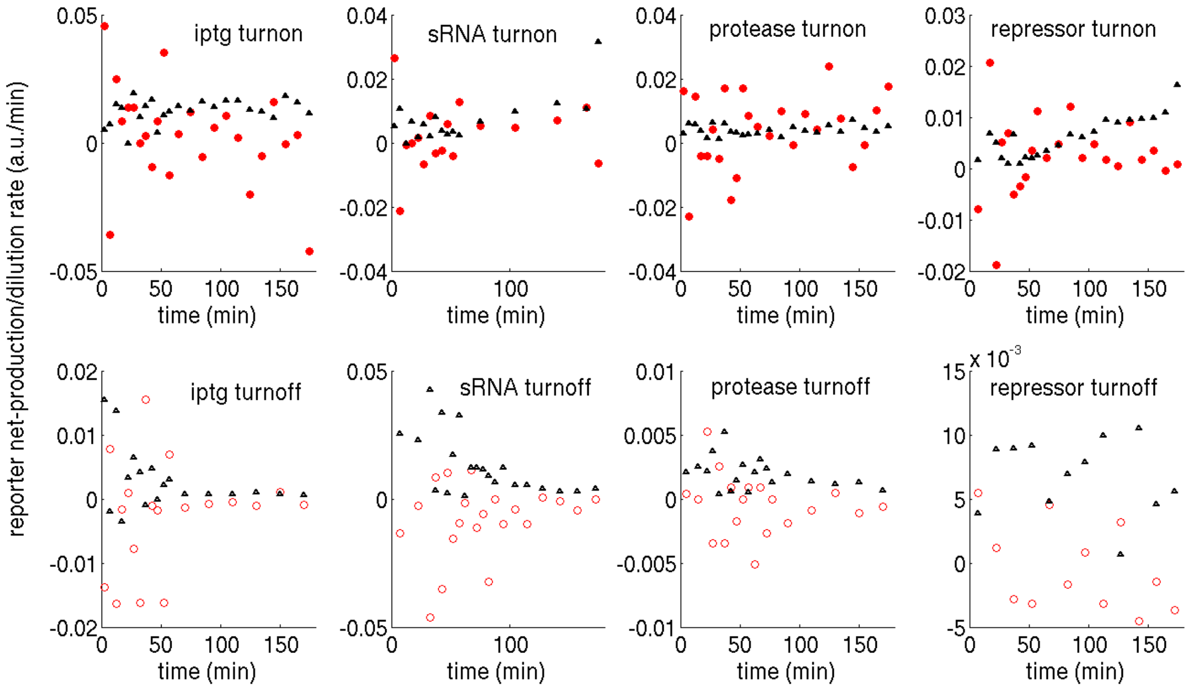


**Figure S1:** Plots show estimates of the net production rate ($p_i \sim dR/dt$, red circles) and dilution rate ($d_i \sim \gamma_{div}R$, black triangles) of the reporter as a function of time for the four experiments shown in the large panels of Figure 2 in the main text. To avoid clutter, the top (turn-on) panels show these quantities only for the case where inducer molecule was present, and bottom (turn-off) panels show only the case where the inducer molecule was absent. The estimation was done as follows: $p_i = (R_{i+1}\text{-R}_i)/(t_{i+1}\text{-}t_i)$ and $d_i = (\ln(O_{i+1})\text{-}\ln(O_i))/(t_{i+1}\text{-}t_i)$, for $i = 1,2,...,\text{D-1}$, where $R_i$, $O_i$ are respectively the reporter level and OD measurement for the D timepoints in each experiment: $t_i$, $i = 1,2,...,\text{D}$. $p_i$ and $d_i$ were plotted vs. time points $t'_i = (t_i\text{-}t_{i+1})/2$.

## Fitting the model to a subset of the data

Figure 2 of the main text shows the result of fitting the model to the entire data set, using the procedure described in *Material & Methods*. Applying the same procedure to only the four experiments shown in the large panels in Figure 2 results in the following fitted values for the 8 parameters that are common to all experiments (not counting the two parameters fixed by the IPTG steady-state experiment, $\tilde{\beta}_R$and $h_L$ ): $\tilde{\delta}_S\approx$ 0.00075/min, $\tilde{\beta}_L\approx$ 0.12, $\tilde{\alpha}_S\approx$1519.9/min, $\tilde{\alpha}_P\approx$ 0.03/min, $\tilde{\alpha}_T\approx$ 1.01/min, $\tilde{k}_{Ltl}\approx$ 0.2/min, $\tilde{I}\approx$19.5, and $h_T\approx$ 1.31.

The dynamical regime that these values place the regulators in is the same as that obtained from the full fit presented in the main text: The repressor has low cooperativity, close to saturating production rate and a low but not insignificant leak; the sRNA has a low γ value and high α value (see reference 1, and section ”Interpreting rescaled parameters” below); the protease has a low (rescaled) production rate; and IPTG is present at saturating levels. Thus, using these parameter values instead of the ones employed in Figure 2, would not affect any of our conclusions. However, obtaining an additional parameter set by this “limited fit” exercise provides information about which parameters are likely to more more or less constrained by the data, that is, which have more leeway in the model fitting.

We further checked that keeping these 8 parameters fixed (and the 2 parameters obtained from the IPTG steady-state fit), and allowing $\tilde{k}_{Rtl}\approx$ and initial values of mRNAs and proteins to vary, results in a fit to all data (see Figure S2) that is as good as the fit in Figure 2. Together these two fits and their comparison provide adequate evidence that our biologically motivated model is sufficient to describe the experimental data.

A

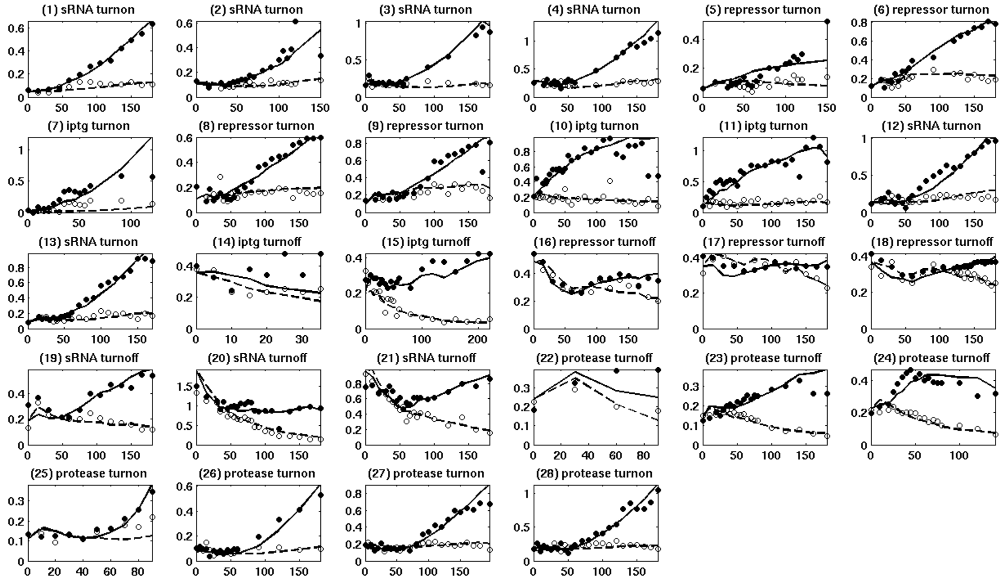

B

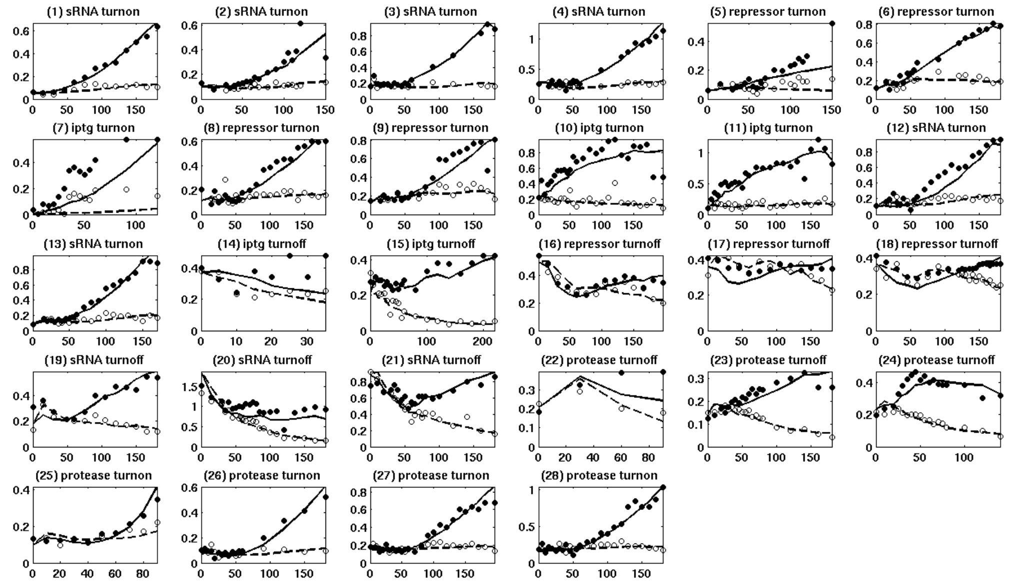


**Figure S2**: (A) Fit to the full data set. This is the same fit as shown in Figure 2 of the main text. The parameters that are common to all experiments have the following values: $\tilde{\delta}_S \approx 0.001$/min, , $\tilde{\beta}_L \approx 0.18$, $\tilde{\alpha}_S \approx 1739.3$/min, $\tilde{\alpha}_P \approx 0.03$/min, $\tilde{\alpha}_T \approx 0.99$/min, $\tilde{k}_{Ltl} \approx 0.2$/min, $\tilde{I} \approx 23.45$, and $h_T \approx 1.016$..
(B) "Limited fit". Here, the above parameters were first determined by fitting only to experiments 3, 8, 10, 15, 17, 20, 23 and 28 (the ones shown in the large panels of Figure 2). This resulted in parameter values: $\tilde{\delta}_S \approx 0.00075$/min, , $\tilde{\beta}_L \approx 0.12$, $\tilde{\alpha}_S \approx 1519.9$/min, $\tilde{\alpha}_P \approx 0.03$/min, $\tilde{\alpha}_T \approx 1.01$/min, $\tilde{k}_{Ltl} \approx 0.2$/min, $\tilde{I} \approx 19.5$, and $h_T \approx 1.31$. Keeping these parameters fixed, as well as $\tilde{\beta}_R$ and $h_{L,}$ the model was then fitted to the rest of the data allowing $\tilde{k}_{Rtl}$ and initial conditions of mRNAs and proteins to vary, which resulted in the plots shown.

**Table S1: Fitted parameter values for $\widetilde{k}_{Rtl}$ and the levels of the mRNAs and proteins at time zero, which were different for each experiment.**

| Experiment | LacI $L'_{tot}$ | Reporter *R'* | sRNA *S'* | Protease *P'* | Repressor *T'* | LacI mRNA $L'_m$ | Reporter mRNA $R'_m$ | $\widetilde{k}_{Rtl}$ (-ara) | $\widetilde{k}_{Rtl}$ (+ara) |
|---|---|---|---|---|---|---|---|---|---|
| 1 | 2.16 | 0.04 | | | | 28.99 | 0.04 | 0.05 | 0.02 |
| 2 | 0.09 | 0.11 | | | | 53.53 | 0.10 | 0.08 | 0.02 |
| 3 | 0.22 | 0.15 | | | | 48.82 | 0.33 | 0.08 | 0.04 |
| 4 | 1.50 | 0.29 | | | | 39.25 | 0.00 | 0.14 | 0.04 |
| 5 | 2.40 | 0.05 | | | | 0.64 | 0.33 | 0.02 | 0.02 |
| 6 | 0.50 | 0.11 | | | | 7.27 | 0.07 | 0.07 | 0.07 |
| 7 | 11.49 | 0.00 | | | | 150.07 | 0.00 | 0.19 | 0.18 |
| 8 | 1.66 | 0.11 | | | | 10.68 | 0.06 | 0.06 | 0.06 |
| 9 | 1.76 | 0.09 | | | | 11.80 | 0.20 | 0.11 | 0.09 |
| 10 | 0.03 | 0.22 | | | | 6.68 | 0.16 | 0.04 | 0.03 |
| 11 | 0.08 | 0.12 | | | | 22.52 | 0.04 | 0.07 | 0.03 |
| 12 | 0.15 | 0.14 | | | | 72.86 | 0.14 | 0.16 | 0.04 |
| 13 | 0.91 | 0.09 | | | | 41.18 | 0.13 | 0.10 | 0.04 |
| 14 | 1.91 | 0.36 | | | | 126.79 | 0.05 | 0.02 | 0.02 |
| 15 | 0.47 | 0.21 | | | | 40.66 | 1.53 | 0.01 | 0.01 |
| 16 | 4.94 | 0.49 | | | 0.29 | 0.24 | 0.17 | 0.06 | 0.03 |
| 17 | 0.35 | 0.35 | | | 6.09 | 2.42 | 0.21 | 0.03 | 0.03 |
| 18 | 4.94 | 0.37 | | | 3.83 | 0.19 | 0.15 | 0.04 | 0.03 |
| 19 | 0.49 | 0.19 | 123.65 | | | 16.46 | 1.01 | 0.04 | 0.02 |
| 20 | 0.20 | 1.94 | 789.27 | | | 4.30 | 0.08 | 0.02 | 0.02 |
| 21 | 1.70 | 0.96 | 1242.88 | | | 12.93 | 0.60 | 0.02 | 0.02 |
| 22 | 0.98 | 0.24 | | 0.39 | | 112.92 | 2.69 | 0.02 | 0.02 |
| 23 | 0.46 | 0.14 | | 1.90 | | 9.22 | 0.79 | 0.02 | 0.01 |
| 24 | 0.32 | 0.22 | | 26.90 | | 49.84 | 0.53 | 0.04 | 0.01 |
| 25 | 0.15 | 0.11 | | 0.55 | | 180.22 | 0.04 | 0.35 | 0.30 |
| 26 | 0.55 | 0.07 | | | | 60.20 | 0.12 | 0.05 | 0.04 |
| 27 | 1.27 | 0.18 | | | | 27.00 | 0.13 | 0.09 | 0.05 |
| 28 | 0.05 | 0.17 | | | | 18.51 | 0.10 | 0.08 | 0.06 |

**Rescaling of model equations.**

The complete set of equations that we use to model regulation by IPTG, repressor, sRNA and protease, are:

$$\frac{dL_m}{dt} = \frac{\alpha_L + \beta_L (T/K_T)^{h_T}}{1 + (T/K_T)^{h_T}} - \gamma_{Lm} L_m - \gamma_{div} L_m - \delta_S L_m S \tag{18}$$

$$\frac{dL_{tot}}{dt} = k_{Ltl} L_m - \gamma_{div} L_{tot} - \delta_P L_{tot} P \tag{19}$$

$$\frac{dS}{dt} = \alpha_S - \delta_S L_m S - \gamma_{div} S \tag{20}$$

$$\frac{dP}{dt} = \alpha_P - \gamma_{div} P \tag{21}$$

$$\frac{dT}{dt} = \alpha_T - \gamma_{div} T \tag{22}$$

$$\frac{dR_m}{dt} = \frac{\alpha_R + \beta_R (L/K_L)^{h_L}}{1 + (L/K_L)^{h_L}} - \gamma_{Rm} R_m - \gamma_{div} R_m \tag{23}$$

$$\frac{dR}{dt} = k_{Rtl} R_m - \gamma_{div} R \tag{24}$$

$$L = L_{tot}/\tilde{I} \tag{25}$$

The parameters $\alpha$, $\beta s$, have units of concentration/time, $ks$ have units of 1/time, $\gamma s$ have units of 1/time, $\delta s$ have units of 1/(concentration*time), $Ks$ have units of concentration, and $hs$ are dimensionless.

As mentioned in the main text, values for all the parameters in the above equations cannot be independently determined by fitting to data, because the data consist only of reporter concentrations, and not concentrations of the other components of the system. The combinations of parameters that can be determined by fitting are obtained by first rescaling the variables as follows: $L'_{tot} = \frac{L_{tot}}{\tilde{I} K_L}$, where $\tilde{I} = \frac{1+(\frac{I}{K_I})^{h_I}}{1+\beta_I(\frac{I}{K_I})^{h_I}}$,

$S' = S\frac{\gamma_{Lm}}{\alpha_L}$, $P' = P\sqrt{\frac{\gamma_P}{\alpha_P}}$, $T' = \frac{T}{K_T}$, $L'_m = L_m\frac{\gamma_{Lm}}{\alpha_L}$, $R'_m = R_m\frac{\gamma_{Rm}}{\alpha_R}$, and $R' = R/\lambda$, where λ is a mutliplicative factor used to convert the measurements of β-gluc activity to real concentrations of β-gluc per cell. All these new variables are now dimensionless.

Using the rescalings in the above equations we get:

$$\frac{dL'_m}{dt} = \gamma_{Lm}\frac{1+(\beta_L/\alpha_L)(T')^{h_T}}{1+(T')^{h_T}} - \gamma_{Lm}L'_m - \gamma_{div}L'_m - \frac{\delta_S\alpha_L}{\gamma_{Lm}}L'_mS' \quad (26)$$

$$\frac{dL'_{tot}}{dt} = \frac{\alpha_L k_{Ltl}}{\tilde{I}K_L\gamma_{Lm}}L'_m - \gamma_{div}L'_{tot} - \sqrt{\alpha_P\delta_P}L'_{tot}P' \quad (27)$$

$$\frac{dS'}{dt} = \frac{\alpha_S\gamma_{Lm}}{\alpha_L} - \frac{\delta_S\alpha_L}{\gamma_{Lm}}L'_mS' - \gamma_{div}S' \quad (28)$$

$$\frac{dP'}{dt} = \sqrt{\alpha_P\delta_P} - \gamma_{div}P' \quad (29)$$

$$\frac{dT'}{dt} = \alpha_T/K_T - \gamma_{div}T' \quad (30)$$

$$\frac{dR'_m}{dt} = \gamma_{Rm}\frac{1+(\beta_R/\alpha_R)(L'_{tot})^{h_L}}{1+(L'_{tot})^{h_L}} - \gamma_{Rm}R'_m - \gamma_{div}R'_m \quad (31)$$

$$\frac{dR'}{dt} = \frac{k_{Rtl}\alpha_R}{\lambda\gamma_{Rm}}R'_m - \gamma_{div}R' \quad (32)$$

We can then define new rescaled parameters:

$\tilde{\beta}_L = \beta_L/\alpha_L$, $\tilde{\alpha}_S = \frac{\alpha_S\gamma_{Lm}}{\alpha_L}$, $\tilde{\delta}_S = \frac{\alpha_L\delta_S}{\gamma_{Lm}}$, $\tilde{\alpha}_P = \sqrt{\alpha_P\delta_P}$, $\tilde{\alpha}_T = \alpha_T/K_T$, $\tilde{k}_{Ltl} = \frac{\alpha_L k_{Ltl}}{\gamma_{Lm}K_L}$, $\tilde{k}_{Rtl} = \frac{k_{Rtl}\alpha_R}{\lambda\gamma_{Rm}}$, $\tilde{\beta}_R = \beta_R/\alpha_R$.

These are the parameter combinations that can be determined by fitting to data (see Fig. 2), and in terms of these, the final equations used to fit the data are:

$$\frac{dL'_m}{dt} = \gamma_{Lm}\frac{1+\tilde{\beta}_L(T')^{h_T}}{1+(T')^{h_T}} - \gamma_{Lm}L'_m - \gamma_{div}L'_m - \tilde{\delta}_S L'_m S' \quad (33)$$

$$\frac{dL'_{tot}}{dt} = \frac{\tilde{k}_{Ltl}}{\tilde{I}}L'_m - \gamma_{div}L'_{tot} - \tilde{\alpha}_P L'_{tot}P' \quad (34)$$

$$\frac{dS'}{dt} = \tilde{\alpha}_S - \tilde{\delta}_S L'_m S' - \gamma_{div}S' \quad (35)$$

$$\frac{dP'}{dt} = \tilde{\alpha}_P - \gamma_{div}P' \quad (36)$$

$$\frac{dT'}{dt} = \tilde{\alpha}_T - \gamma_{div}T' \quad (37)$$

$$\frac{dR'_m}{dt} = \gamma_{Rm}\frac{1+\tilde{\beta}_R\left(L'_{tot}\right)^{h_L}}{1+\left(L'_{tot}\right)^{h_L}} - \gamma_{Rm}R'_m - \gamma_{div}R'_m \quad (38)$$

$$\frac{dR'}{dt} = \tilde{k}_{Rtl}R'_m - \gamma_{div}R' \quad (39)$$

*Interpreting the rescaled parameters*

The $\tilde{\beta}$s are easy to interpret, they are the relative size of the leak in reporter and *lacI* transcription, and can be directly compared to 1. That is, the leak is small if $\tilde{\beta} \ll 1$ and is large if $\tilde{\beta} \gtrsim 1$. All the other rescaled parameters are easy to interpret in the situation of constant growth rate, i.e. when $\gamma_{div}$ is a constant. In that case, if $\frac{\tilde{\alpha}_T}{\gamma_{div}} \gg 1$ then the repressor is repressing strongly. Similarly, if $\frac{\tilde{\alpha}_P^2}{\gamma_{div}} \gg \gamma_{div}$ then the protease is strongly active. The two relevant parameters to describe sRNA action[1] are: $\gamma \equiv \tilde{\delta}_S\gamma_{Lm}/\gamma_{div}^2$ and $\alpha \equiv \tilde{\alpha}_S/\gamma_{Lm}$. Finally, $\tilde{k}_{Ltl}/\gamma_{div}$ is the maximum level of LacI in units of the dissociation constant of its binding to its operator site. So, if $\tilde{k}_{Ltl}/\gamma_{div} \gg 1$ then there is enough LacI to repress the reporter strongly.

**Table S2. Bacterial strains and plasmids used in this study.**

| Strains | Relevant genotype | Source |
|---|---|---|
| MG1655 | F- λ- ilvG- rfb-50 rph-1. | Gift from Sankar Adhya |
| SAH1 | UidR::zeoR-rrnBT1T2-PUV5 | This study |
| SAH317 | SAH1 carrying the engineered *lacI* gene | This study |
| SAH538 | SAH317 araCBAD::$cm^R$. | This study |

| Plasmids | Relevant content | Source |
|---|---|---|
| pSA850 | *rpoC terminator* | [2] |

| | | |
|---|---|---|
| pSEM2027 | promoter cloning cassette | [3] |
| pTH24:TEVsh. | TEV protease | [4] |
| pBAD-qrr2 | pBAD24 carrying the Qrr2 sRNA gene | This study |
| pBAD-$C_{16-3}$ | pBAD24 carrying the 16-3 C repressor gene | This study |
| pBAD-TEVP | pBAD24 carrying the TEV protease gene | This study |
| pBAD24 | pBAD promoter | [5] |
| pBBR1MCS2 | cloning plasmid | [6] |
| pCR2.1-qrr2 | pCR2.1 carrying Qrr2 sRNA gene. | Eurofins MWG gene synthesis. |
| pCR2.1-LacI-sRNA-32BS. | pCR2.1. carrying the Qrr2 binding site from HapR and part of the *lacI* sequence | Eurofins MWG gene synthesis. |
| pSAH21 | pBBR1MCS2 carrying the *rpoC* terminator and the promoter of the *elacI* gene | This study |
| pSAH32 | pSAH21 carrying the *lacI* coding sequence | This study |
| pSAH44 | pSAH32 carrying the qrr2 binding site | This study |
| pSAH47 | pSAH44 encoding a LacI variant which has a TEVP recognition site | This study |
| pEVS141. | template for *lacI* PCR | [7] |
| pkD3 | Chloramphenicol resistance cassette | [8] |
| pkD46 | recombineering functions | [8] |

**Table S3. Oligonucleotides used in the construction of strains and plasmids.**

| **Name** | **Sequence 5'→3'** |
|---|---|
| RIupPL | AAAAGAATTCGGTACCAGCGACAATTGCTTGTGG<u>N</u>CTACAATTGATTGTAGCCCTATAACTCTCCTCA |
| dnPLPst | AAAACTGCAGTCTAGATTCATAAGCTTAGTTGTTGATTGAGCATGAGGAGAGTTATAGGGCTACAATC |
| RIUV5up | AAAAGAATTCTCACTCATTAGGCACCCCAGGCTTTACACTTTATGCTTCCGGCTCGTAT |
| UV5dnPst | TATACTGCAGAAATTGTGAGCGCTCACAATTCCACACATTATACGAGCCGGAAGCATAAAGTGT |
| NsiRpoCTup | ATATAATGCATTGCAGCAAATAACGTAAAAACCCGCT |
| RpocTdnAcc | ACCATATAGGTACCGGATCCCTAAACTCCCCCCAT |
| HindIIILacIup | CAACTAAGCTTGCAAGGATATACCCCTATGAAACCAGTAACGTTATACGATGTCGCAGAGT |
| lacIdnAtXb | ATATATCTAGATATATGACGTCCTCACTGCCCGCTTTCCAGTCGGGAAAC |
| qcTEV100up | CCGCCGCTTTACAGGACTGAAAGTACAGATTTTCTACCATCGACAC |

| | |
|---|---|
| qcTEV100dn | GTGTCGATGGTAGAAAATCTGTACTTTCAGTCCTGTAAAGCGGCGG |
| LacIuprecomb | CTGGGATCAGGAGGAGAAGATCGCCTCTATCGCCGTACCGCGCAGGTAGCTTGCAGTGGGCTTACATGGC |
| LacInydnrecomb | CAGTCGGGAAACCTGTCGTG |
| pBAD24C16-3up | TTTTTAAGCTTACTACCAATATTTTCCGGCGAGAATGAT |
| pBAD24C16-3dn | AAAAATCTAGAAGGAGGAATTCACCATGCATAAAGGGACATTTCACATGAGC |
| ARACDELREV | CCAATTATGACAACTTGACGGCTACATCATTCACTTTTTCTTCCATATGAATATCCTCCTTA |
| ARADDELFWD | CGCGCCATGCTTACGCAGATAGTGTTTATCCAGCAGCGTTTGTGTAGGCTGGAGCTGCTTC |


1. Mitarai, N. *et al.* Dynamic features of gene expression control by small regulatory RNAs. *Proceedings of the National Academy of Sciences* **106**, 10655-10659 (2009).
2. Lewis, D.E. & Adhya, S. In vitro repression of the gal promoters by GalR and HU depends on the proper helical phasing of the two operators. *J Biol Chem* **277**, 2498-504 (2002).
3. Hunziker, A., Tuboly, C., Horvath, P., Krishna, S. & Semsey, S. Genetic flexibility of regulatory networks. *Proc Natl Acad Sci U S A* **107**, 12998-3003 (2010).
4. van den Berg, S., Lofdahl, P.A., Hard, T. & Berglund, H. Improved solubility of TEV protease by directed evolution. *J Biotechnol* **121**, 291-8 (2006).
5. Guzman, L.M., Belin, D., Carson, M.J. & Beckwith, J. Tight regulation, modulation, and high-level expression by vectors containing the arabinose PBAD promoter. *J Bacteriol* **177**, 4121-30 (1995).
6. Kovach, M.E., Phillips, R.W., Elzer, P.H., Roop, R.M., 2nd & Peterson, K.M. pBBR1MCS: a broad-host-range cloning vector. *Biotechniques* **16**, 800-2 (1994).
7. Dunn, A.K., Millikan, D.S., Adin, D.M., Bose, J.L. & Stabb, E.V. New rfp- and pES213-derived tools for analyzing symbiotic Vibrio fischeri reveal patterns of infection and lux expression in situ. *Appl Environ Microbiol* **72**, 802-10 (2006).
8. Datsenko, K.A. & Wanner, B.L. One-step inactivation of chromosomal genes in Escherichia coli K-12 using PCR products. *Proc Natl Acad Sci U S A* **97**, 6640-5 (2000).
